# Topics of Public Interest

**Published:** 1908-08

**Authors:** 


### TOPICS OF PUBLIC INTEREST.

### New York Police to Stop Noises.

### General Bingham Instructs Men on Violation of City Ordinances.

COMMISSIONER Bingham has received daily a batch of mail in which citizens complain bitterly of the unnecessary noise made by vehicles, automobiles and boys playing and running through the streets with tin cans tied to strings. He issued July 18, 1908, a general order instructing the police to suppress all unnecessary noise and to arrest noise makers. The general order reads:

The police force of this city can put a stop to a large proportion of the unnecessary noises which torment the entire population. Not only is it a matter of health and happiness for all concerned that the people of New York should be disturbed at night as little as possible, but there are also thousands of night workers in the city who must sleep in the day time. It is a part of the duty of the police force on patrol, as the general protectors of the public, to see that unnecessary noise is suppressed. Generally speaking, a public nuisance is an act that annoys, injures or endangers the comfort, repose, health or safety of any considerable number of persons.

In cases in which the noises are a violation of the laws or ordinances, arrests will be made. The unnecessary use of horns, sirens, whistles or bells on automobiles or motorcycles is a public nuisance and a violation of Section 385 of the Penal Code. Hand organs in the Borough of Manhattan are allowed to play from 9 a. m. to 7 p. m. All rails, pillars and columns of iron and steel transported through the streets of the city, causing loud noises, is a violation of the ordinance. No person owning or having animals or birds making unnecessary noise is permitted to keep them. It is unfortunately true that the streets constitute the only playground for thousands of children throughout the greater city, but, even so, the exercise of a little common sense will enable the police to distinguish what is purely unnecessary noise.

The police are directed to suppress the shouting of street hawkers of all kinds; all unnecessary shouting and yelling; unnecessary blowing of steamboat or factory whistles; roller skating on the streets or sidewalks to the interruption and interference of traffic; whistling peanut roasters; unnecessary blowing of whistles or horns on motorcycles or automobiles; letting the exhaust escape from motorcycles and automobiles without being properly muffled; blowing horns or bugles or ringing bells by scissors grinders; yelling of the "old clothes" man; yelling of "extras" at night; kicking tin cans on sidewalks; yelling of carriage barkers at theatres and hotels; flat wheels on streetcars; barking dogs; see owners of such dogs and report for action by the Board of Health; commanding officers will instruct each member of their command to see that all ordinances and laws regarding unnecessary noises, both on land and water, are rigidly enforced, and it is made a duty of the precinct captains to inform themselves of the laws and ordinances on the subject of noise and instruct their men.

The police on duty along the river front will report the names of boats, time and places at which unnecessary whistling is done, and get the names and addresses of witnesses for report to the Police Commissioner.

The police are often unjustly criticised, but if, by a display of common sense and judicious energy, they effect any appreciable reduction in unnecessary noise, they will not only deserve but have the thanks and support of the entire community.

